# Subversion of Cellular Autophagosomal Machinery by RNA Viruses

**DOI:** 10.1371/journal.pbio.0030156

**Published:** 2005-04-26

**Authors:** William T Jackson, Thomas H Giddings, Matthew P Taylor, Sara Mulinyawe, Marlene Rabinovitch, Ron R Kopito, Karla Kirkegaard

**Affiliations:** **1**Departments of Microbiology and Immunology, Stanford UniversityStanford, CaliforniaUnited States of America; **2**Department of Molecular, Cellularand Developmental Biology, University of Colorado, Boulder, ColoradoUnited States of America; **3**Biological Sciences, Stanford UniversityStanford, CaliforniaUnited States of America; **4**Pediatrics, Stanford UniversityStanford, CaliforniaUnited States of America; University of Wisconsin-MadisonUnited States of America

## Abstract

Infection of human cells with poliovirus induces the proliferation of double-membraned cytoplasmic vesicles whose surfaces are used as the sites of viral RNA replication and whose origin is unknown. Here, we show that several hallmarks of cellular autophagosomes can be identified in poliovirus-induced vesicles, including colocalization of LAMP1 and LC3, the human homolog of Saccharomyces cerevisiae Atg8p, and staining with the fluorophore monodansylcadaverine followed by fixation. Colocalization of LC3 and LAMP1 was observed early in the poliovirus replicative cycle, in cells infected with rhinoviruses 2 and 14, and in cells that express poliovirus proteins 2BC and 3A, known to be sufficient to induce double-membraned vesicles. Stimulation of autophagy increased poliovirus yield, and inhibition of the autophagosomal pathway by 3-methyladenine or by RNA interference against mRNAs that encode two different proteins known to be required for autophagy decreased poliovirus yield. We propose that, for poliovirus and rhinovirus, components of the cellular machinery of autophagosome formation are subverted to promote viral replication. Although autophagy can serve in the innate immune response to microorganisms, our findings are inconsistent with a role for the induced autophagosome-like structures in clearance of poliovirus. Instead, we argue that these double-membraned structures provide membranous supports for viral RNA replication complexes, possibly enabling the nonlytic release of cytoplasmic contents, including progeny virions, from infected cells.

## Abstract 2

Poliovirus is a well-studied positive-strand RNA virus, and here it is shown to subvert the machinery of the cellular autophagy pathway at several stages in the viral life cycle

## Introduction

Upon infection by poliovirus and many other positive-strand RNA viruses, dramatic changes are rapidly induced in the cellular environment. Poliovirus infection causes the massive rearrangement of intracellular membranes, with double-membraned vesicles 200–400 nm in diameter accumulating in the cytoplasm [1,2]. Immunoelectron microscopy has revealed that the cytoplasmic surfaces of these membranous vesicles are the sites of viral RNA replication [3,4,5] and, indeed, all known positive-strand RNA viruses of eukaryotic cells replicate their RNA on cytoplasmic membranes. It is thought that one function for membrane localization of viral RNA replication proteins is to promote their oligomerization [6,7].

It has been noted previously that several of the features displayed by the vesicles induced during poliovirus infection [1,2] are known to be shared with cellular membranous structures termed autophagosomes. During cellular autophagy, cells break down cytoplasmic proteins and organelles within autophagosomes, double-membraned structures that become degradative upon maturation. Originally identified as a process induced by cellular starvation, autophagy is now appreciated as a cellular response to a variety of stimuli, including hormone treatment, and is a feature of normal development in several organisms (reviewed in [8]). In mammalian cells, two chemical inducers of autophagy are tamoxifen [9,10] and rapamycin [11,12,13]. Cells that express the estrogen receptor respond to tamoxifen treatment by accumulating large amounts of autophagosomes [9,10]. Rapamycin inhibits the function of the mammalian target of rapamycin, a known repressor of the autophagic pathway (reviewed in [14]). In yeast, formation and maturation of autophagosomes requires the functions of many genes (reviewed in [15]). Human homologs of several of the yeast autophagy genes have been recently identified, including LC3, the human homolog of Atg8p, and it is likely that much of the autophagic pathway is conserved [16,17,18].

In mammalian cells, the formation and maturation of autophagosomes involves the stepwise acquisition of proteins from disparate cellular compartments (reviewed in [19,20,21]). Nascent autophagosomes form either de novo [22,23] or from the endoplasmic reticulum (ER) [24,25] and comprise cellular cytoplasm surrounded by two lipid bilayers that fuse from a C-shaped intermediate. Although relatively protein-poor [22,26], they have been shown to contain a modified, lipidated form of LC3 [16]. As maturation proceeds, the late autophagosome acquires LAMP1 before lysosomal fusion [16,25,27,28], which defines the “mature autolysosome.” Mature autolysosomes are no longer surrounded by double membranes and, having degraded the inner membrane and much of the cytosolic contents, become electron-dense [27,29]. Various intermediates among these stages have also been visualized, including incompletely fused double membranes and incompletely degraded inner membranes [23,24,25].

Although poliovirus-induced vesicles display several hallmarks of autophagosomes, other origins for the poliovirus-induced vesicles have been suggested [30,31,32]. Poliovirus RNA replication is known to be inhibited by brefeldin A, which has led to the suggestion that the virally induced vesicles might derive from the COPI pathway, a known target of brefeldin A [32,33,34]. However, this would be inconsistent with the apparent origin of the poliovirus-induced vesicles from the ER: many images consistent with the budding of the poliovirus-induced vesicles directly from the ER have been reported [3,31]. Furthermore, poliovirus proteins 2BC and 3A induce the formation of vesicles that are biochemically and ultrastructurally similar to those formed in poliovirus-infected cells [35]. The 3A protein and 2C, a proteolytic product of 2BC, localize to the ER when expressed in isolation [35,36]. Therefore, it is likely that poliovirus proteins 3A and 2BC, or larger precursors, localize to the ER early in infection and subsequently promote vesiculation from ER membranes.

Poliovirus-induced vesicles have been shown to contain human COPII proteins Sec13p and Sec31p early in their formation, leading to the hypothesis that they are modified COPII vesicles, subverted from the anterograde transport pathway [31]. Although this is an attractive suggestion, it is incongruent with other findings concerning the poliovirus-induced vesicles: their frequently observed double-membraned morphology, their cytosolic contents, and the abundance of components from throughout the secretory pathway, including LAMP1, a marker of late endosomes and lysosomes [1,2,35]. Furthermore, in *Saccharomyces cerevisiae,* components of the COPII pathway, encoded by the *SEC12, 16, 23,* and *24* genes, are known to be required for autophagy [37,38,39], making it possible that the Sec13p and Sec31p proteins could have other functions in addition to their known roles in anterograde traffic. The size of the poliovirus-induced vesicles, at 200–400 nm in diameter, does not immediately suggest whether they are related to the COPII or autophagosomal pathways: typical diameters of COPII vesicles and autophagosomes are 50–100 nm [40,41] and 500–1,000 nm, respectively [24,25].

Finally, the idea that poliovirus infection induces autophagosome formation to facilitate viral growth is surprising in view of existing data that autophagy is an effective antimicrobial host response in many cases (reviewed in [42,43]). During herpes virus infection, there is good correlation between the induction of autophagy and the presence of effective antiviral responses. The process of autophagy is thought to be destructive to herpes virus because mutant viruses that fail to inhibit autophagy and other antiviral responses showed large decreases in yield [44]. During *Streptococcus* infection, genetic disruption of the autophagy pathway in the infected host cells resulted in increased bacterial yield, consistent with a role for autophagy in bacterial clearance [45].

Specific markers for autophagosome formation have become available, facilitating the identification of membranes derived from the autophagic pathway (reviewed in [43,46]). Here, we explore the role of several constituents of autophagosome machinery in poliovirus- and rhinovirus-infected cells by monitoring: the presence of autophagosomal protein LC3 in virally induced vesicles, the acquisition of colocalization of LC3 and LAMP1 in virally infected cells, the viral induction of punctate structures that stain with monodansylcadaverine (MDC), and the effects of perturbing the autophagosomal pathway pharmacologically and via RNA interference on intracellular and extracellular virus yield. Our data support the hypothesis that poliovirus, and likely the closely related rhinovirus, induce the formation of autophagosome-like structures to serve as the membrane scaffolds for RNA replication. We further suggest that double-membraned vesicles, by forming a luminal cytoplasmic compartment, may facilitate prelytic viral exit from infected cells.

## Results

### LC3, a Marker of Cellular Autophagosomes, Colocalizes with Proteins of the Poliovirus RNA Replication Complex

**Figure 1:**
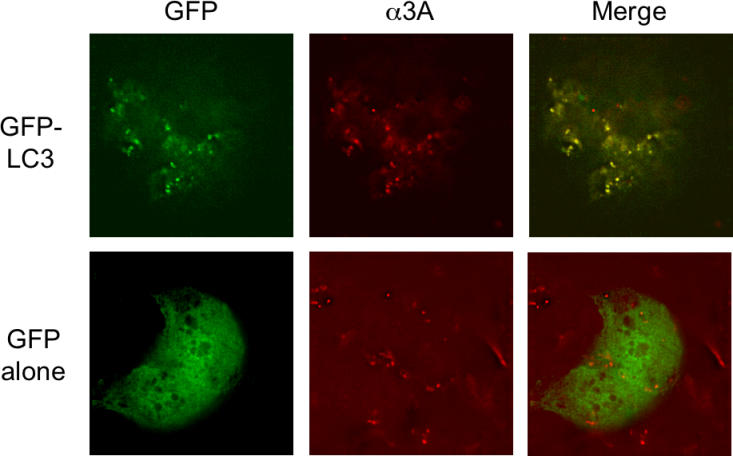
Simultaneous Visualization of GFP-LC3 and Poliovirus Protein 3A (Red) MCF-7 cells were transfected either with a plasmid that expresses a GFP-LC3 fusion protein or GFP alone as indicated. Forty-eight hours posttransfection, cells were infected with poliovirus, then fixed and stained using a monoclonal antibody to poliovirus 3A protein and a rhodamine-conjugated secondary antibody.

To ask whether the membranes on which poliovirus RNA replication complexes assemble contain constituents of autophagosomes, we monitored the localization of both LC3, a specific marker of autophagosomes, and 3A, a critical component of the poliovirus RNA replication complex, in infected cells. LC3 was expressed via DNA transfection as an amino-terminal fusion with green fluorescent protein (GFP). As can be seen in Figure 1, the punctate GFP–LC3 signal colocalized with that of poliovirus RNA replication protein 3A, as visualized by immunofluorescence. No colocalization with poliovirus 3A protein was seen when GFP was not fused to LC3. Therefore, the membranous structures to which 3A and other components of the poliovirus RNA replication complex localize [3] can recruit LC3.

### LAMP1 and LC3 Colocalize in Both Tamoxifen-Treated, Rapamycin-Treated, and Poliovirus-Infected Cells

**Figure 2:**
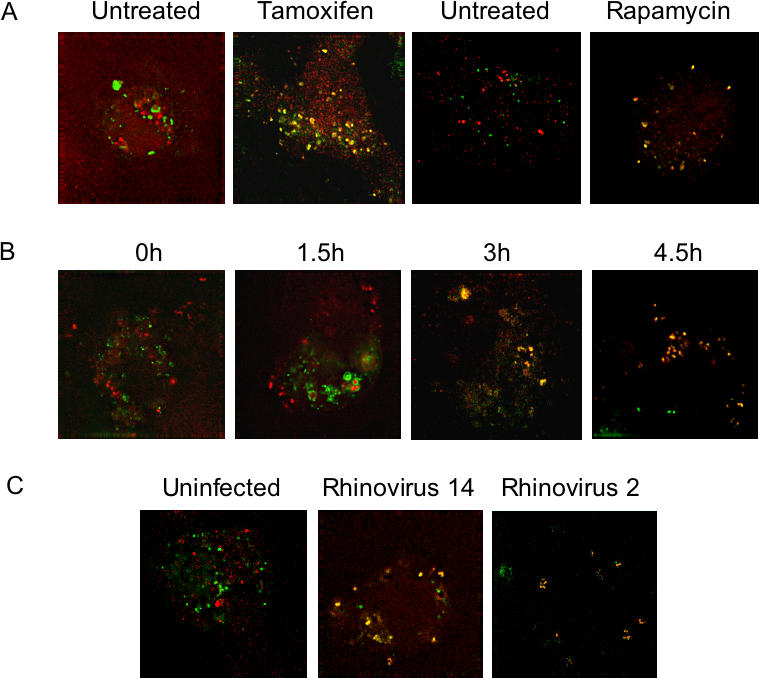
Simultaneous Visualization of GFP-LC3 and Resident Lysosomal Protein LAMP1 (Red) during Autophagic Induction, Poliovirus Infection, and Rhinovirus 14 Infection (A) MCF-7 cells were transfected with a plasmid that expresses GFP-LC3 fusion protein and treated with 10 μM tamoxifen in DMSO/EtOH or with DMSO/EtOH alone as indicated. H1-HeLa cells were transfected with the GFP-LC3-expressing plasmid and treated with rapamycin for 5 h or left untreated as indicated. (B) A time course of poliovirus infection was performed in GFP-LC3-transfected MCF-7 cells, followed by visualization of LAMP1 and GFP-LC3 localization. Infections were with Mahoney type 1 poliovirus at a multiplicity of infection (MOI) of 50 PFU/cell for 5 h at 37 °C. (C) H1 HeLa cells were transfected with an LC3-GFP-expressing plasmid and either mock-infected or infected with human rhinoviruses as indicated at 50 infectious units/cell for 6 h at 33.5 °C.

In nonautophagic cells, LC3, originally identified as a microtubule-associated protein [47], and LAMP1, a marker of late endosomes and lysosomes, do not colocalize. However, during autophagy, LC3 has been shown to colocalize with lysosomal protein LAMP1, defining the maturation of the nascent autophagosome to the autophagosome [28,48,49]. This process can be seen in Figure 2A, in which LC3 and LAMP1 were shown to colocalize almost completely in MCF-7 cells upon treatment with tamoxifen, a known inducer of autophagy. Similarly, rapamycin treatment of HeLa cells led to the colocalization of GFP–LC3 and LAMP-1, while they remained distinct in untreated cells (Figure 2A). By 3 h postinfection with poliovirus, the LAMP1 and GFP–LC3 signals in HeLa cells also began to colocalize (Figure 2B); this time corresponds to the beginning of viral RNA synthesis in infected HeLa cells [50]. By 4.5 h postinfection, the LAMP1 and GFP–LC3 signals had merged almost completely (Figure 2B). The colocalization of GFP–LC3 and LAMP1 characteristic of autophagosome formation was also seen in cells infected with either of two different serotypes of human rhinovirus (Figure 2C).

### Simultaneous Expression of Poliovirus Proteins 2BC and 3A Induces the Colocalization of GFP–LC3 and LAMP1

**Figure 3:**
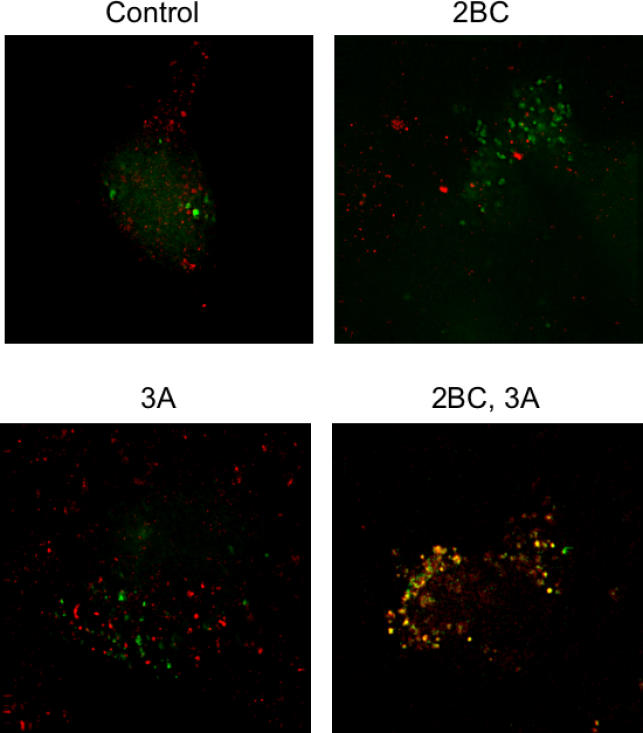
Simultaneous Visualization of GFP-LC3 and Resident Lysosomal Protein LAMP1 (Red) in Cells that Express Poliovirus 2BC and 3A Proteins 293T cells were transfected with vectors expressing 2BC, 3A, or both and a GFP-LC3 expressing vector for 48 h at 37 °C. Nonexpressing vector DNA was used to ensure that all transfections contained the same amount of DNA.

Expression of poliovirus proteins 2BC and 3A in isolation can induce the formation of double-membraned vesicles that display biochemical and ultrastructural similarity to those formed in poliovirus-infected cells [35]. However, expression of poliovirus protein 2BC alone has been shown to induce the formation of single-membraned vesicles in both mammalian cells and S. cerevisiae [4,35,51,52]. Expression of poliovirus 3A protein in isolation, on the other hand, reduces the rate of ER-to-Golgi traffic and distends ER membranes [36,53]. To test whether 2BC, 3A, or both were sufficient to induce colocalization of GFP–LC3 and LAMP1 as observed in poliovirus-infected cells, proteins 2BC and 3A were expressed singly or in combination in 293T cells in the presence of GFP–LC3. As shown in Figure 3, colocalization of GFP–LC3 and LAMP1 was not observed in control cells or in cells that expressed either 2BC or 3A in isolation. However, coexpression of 2BC and 3A caused GFP–LC3 and LAMP1 to colocalize (Figure 3A), as was seen in cells infected with poliovirus or rhinoviruses treated with tamoxifen or treated with rapamycin (see Figure 2).

### Poliovirus Infection and Autophagic Induction Induce Punctate MDC Staining

**Figure 4:**
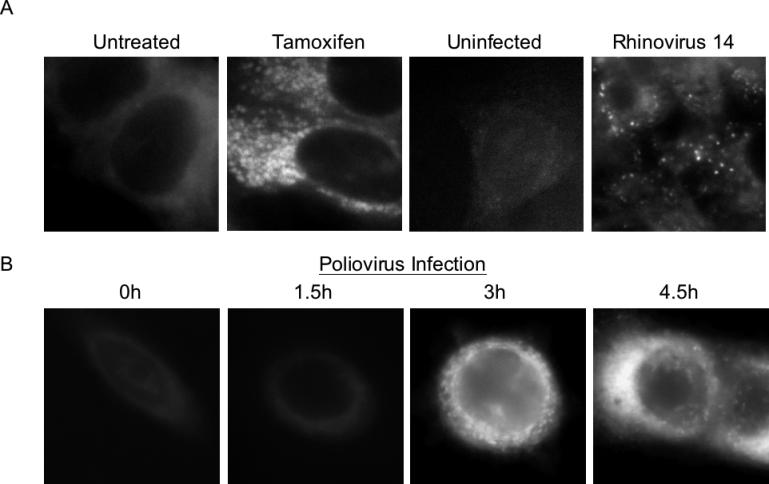
MDC Staining of MCF7 Cells upon Tamoxifen Treatment, Rhinovirus 14 Infection, or Poliovirus Infection (A) Cells that were treated with 10 μM tamoxifen in DMSO/EtOH or with DMSO/EtOH alone for 48 h at 37 °C, or that were mock-infected or infected with human rhinovirus 14 as in Figure 3, were incubated with 100 μM MDC, fixed, and visualized by deconvolution microscopy in the UV channel as described in Materials and Methods. (B) Cells were infected with poliovirus at an MOI of 50 PFU/cell for 0, 1.5, 3, or 4.5 h at 37 °C; for each time point, MDC incubation was begun 1 h prior to fixation and visualization.

To examine further whether the membranes induced during poliovirus infection display additional characteristics of autophagosomes, we employed fluorescent staining with MDC. Under specific fixation conditions, MDC is retained in autophagosomal membranes [54,55]. As shown in Figure 4, treatment of MCF7 human breast tumor cells with tamoxifen caused the accumulation of punctate structures that retained staining with MDC after fixation. Similar punctate staining was observed in cells infected for 5 h with rhinovirus 14 (Figure 4A) or for 3 h or more with poliovirus (see Figure 4B).

**Figure 5:**
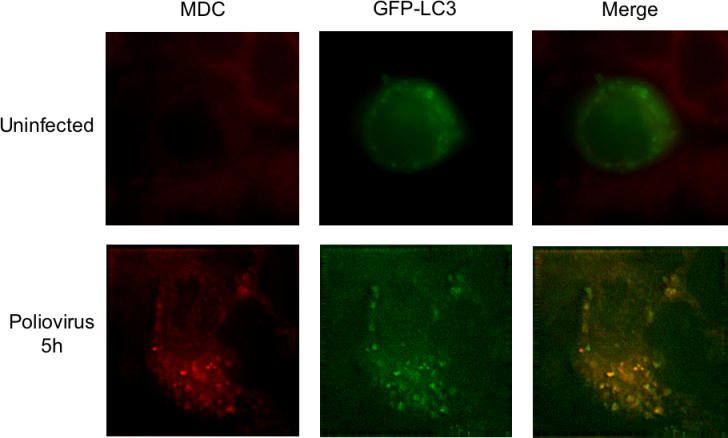
Simultaneous Visualization of GFP-LC3 Localization and MDC Staining (Red) in Uninfected and Poliovirus-Infected Cells Forty-eight hours posttransfection of MCF-7 cells with a plasmid that expresses a GFP-LC3 fusion protein, cells were mock-infected or infected with poliovirus at an MOI of 50 PFU/cell for 5 h at 37 °C. After fixation, deconvolution microscopy was used to visualize fluorescence from both the MDC and GFP fluorescent molecules. MDC incubation was begun 1 h prior to fixation and visualization.

To test the hypothesis that the MDC-stained structures observed in Figure 4 were the same as the GFP–LC3-containing structures observed in Figure 2, we monitored the localization of GFP–LC3 with MDC. As a live stain, MDC is a poor marker for autophagosomes, because both autophagic membranes and lysosomes are visualized [46]. However, if cells are subjected to a defined fixation protocol after staining with MDC, the dye can be removed from single-membraned, but not double-membraned, vesicles [54,55]. As shown in Figure 5, good colocalization of MDC and LC3 was observed in poliovirus-infected cells, and no evidence of the punctate MDC staining expected of lysosomal membranes could be seen in uninfected cells.

### Compounds That Stimulate and Inhibit Cellular Autophagy Affect Yield of Intracellular Poliovirus

**Figure 6:**
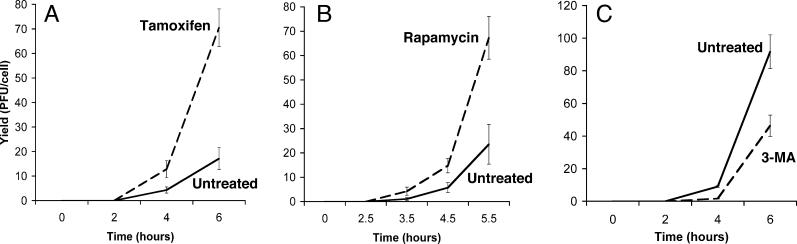
Intracellular Viral Yields from Poliovirus Infections Performed in the Presence of Pharmacological Inducers and Inhibitors of Autophagy (A) H1 HeLa cells were treated with 10 μM tamoxifen in DMSO/EtOH or in DMSO/EtOH alone for 48 h at 37 °C. Cell numbers were determined, and triplicate plates were infected with poliovirus at an MOI of 0.1 PFU/cell for the times indicated. (B) H1 HeLa cells were treated with 50 nM rapamycin in DMSO/EtOH or DMSO/EtOH alone for 3 h before infection with poliovirus as in (A). (C) H1 HeLa cells were treated with 10 mM 3-methyladenine in DMSO/EtOH or DMSO/EtOH alone for 3 h before infection with poliovirus as in (A). Viral yields were determined by plaque assay in H1-HeLa cells and expressed as PFU/cell.

To determine whether the autophagosome-like membranes induced during poliovirus infection perform an antiviral function or facilitate viral replication, we tested the effect on poliovirus yield of pretreating H1–HeLa cells with either tamoxifen or rapamycin, known inducers of autophagy. Yield of intracellular virus increased approximately 4-fold when cells were pretreated with tamoxifen, and 3-fold upon pretreatment with rapamycin (Figure 6A and 6B). Conversely, when cells were treated with 3-methyladenine, a pharmacological inhibitor of autophagy [56], yield of intracellular virus was decreased (Figure 6C). Therefore, during poliovirus infection, the activity of the autophagy pathway correlates with productive viral replication, not with viral destruction.

### A Potential Role for Autophagosome-Like Membranes in Extracellular Delivery of Viruses

**Figure 7:**
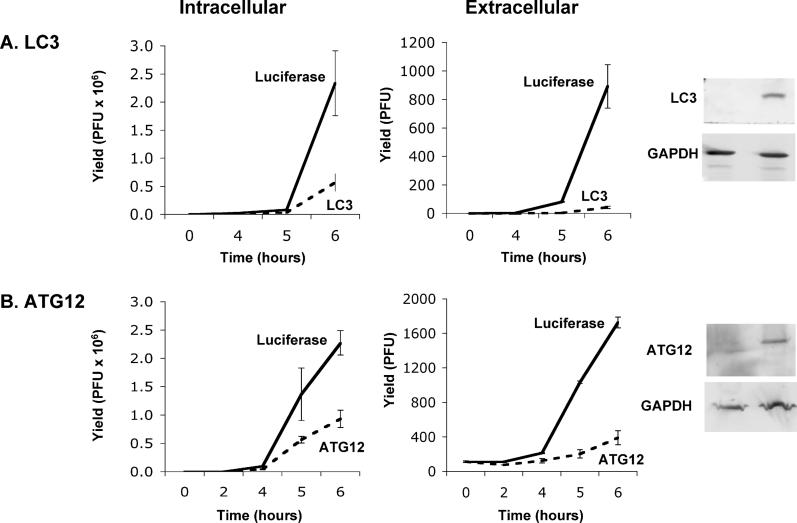
Intracellular and Extracellular Viral Yields from Poliovirus Infections of Cells Treated with Small RNA Duplexes to Reduce the Intracellular Concentrations of LC3 and Atg12p Proteins (A) H1 HeLa cells (1 × 10^6^) were transfected with 12.5 pm each of eight different RNA duplexes targeted to the *LC3A* and *LC3B* mRNAs, or with 100 pm of an RNA duplex targeted to firefly luciferase for 24 h at 37 °C (see Materials and Methods). Cell numbers were determined and triplicate plates were infected with poliovirus at an MOI of 0.1 PFU/cell for the times indicated. Viral yields were determined by plaque assay in H1-HeLa cells and expressed as PFU/cell for the intracellular virus, and as total PFU/plate for the extracellular virus. The relative abundance of LC3 protein in the cells incubated with the control and LC3-targeted RNAi molecules was determined by immunoblot using polyclonal antibodies directed against LC3 and GAPDH. (B) H1 HeLa cells were transfected with 25 pm each of four RNA duplexes targeted to *ATG12* mRNA, or with 100 pm of an RNA duplex targeted to firefly luciferase, for 24 h at 37 °C (see Materials and Methods). Poliovirus infections were performed as in (A). The relative abundance of Atg12p protein was determined by immunoblot using antibodies against human Atg12p and GAPDH.

To extend the studies with pharmacological agents, we tested the effect of reducing the amount of intracellular autophagy proteins LC3 and Atg12p on poliovirus yield, using double-stranded RNA oligonucleotides designed to target their mRNAs for destruction by RNA interference. Pools of double-stranded oligonucleotides were synthesized to target *ATG12* mRNA, and to target both *LC3A* and *LC3B* mRNAs (see Materials and Methods). As shown in Figure 7, the intracellular abundance of Atg12p and LC3 proteins could be reduced to 70% and 90%, respectively, of their abundance in cells treated with control double-stranded small interfering RNAs (siRNAs) known to target firefly luciferase mRNA. These reductions in Atg12p and LC3 protein concentration resulted in 3-fold and 4-fold reductions, respectively, in yields of intracellular poliovirus. Even stronger effects of the RNA interference (RNAi)-mediated reduction in autophagy proteins on viral yield were observed, however, when the yields of extracellular virus were examined. The effects of RNAi against *ATG12* and *LC3* on extracellular virus were 9-fold and 20-fold, respectively (Figure 7). Two potential mechanisms for this preferential reduction in extracellular, as opposed to intracellular virus, seemed possible. The first possibility was that the RNAi-mediated reductions in the intracellular concentrations of Atg12p and LC3 protein reduced cell lysis and therefore lytic release of virus; links between autophagy and apoptosis have been reported (reviewed in [57,58]). The possibility of decreased cellular susceptibility to lysis upon RNAi treatment is difficult to exclude. However, early cell lysis in the presence of reduced Atg12p or LC3 concentrations seemed unlikely because the amounts of virus released were very small, and the observed reductions in extracellular virus release were seen at early time points, before lysis was expected.

**Figure 8:**
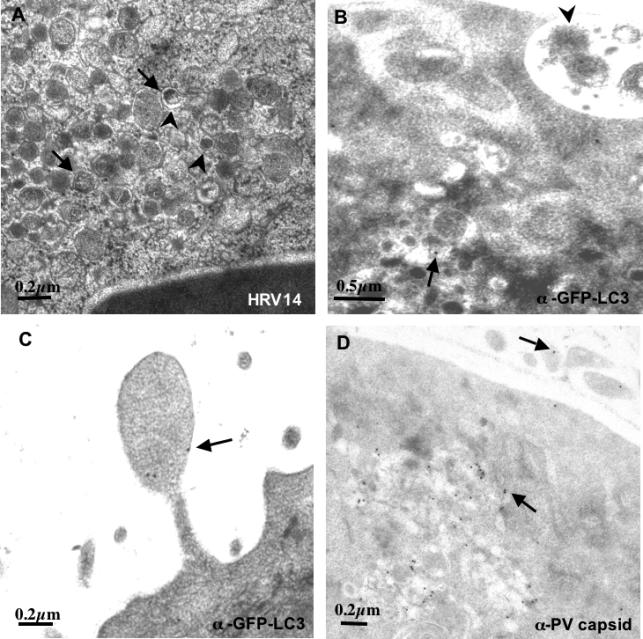
Ultrastructure of H1-HeLa Cells Infected with Human Rhinovirus 14 (HRV14) and Immunoelectron Microscopy of Cells Infected with Poliovirus (A) Cells were infected with rhinovirus 14 as in Figure 3 and prepared for electron microscopy by high-pressure freezing. Examples of readily discernable double lipid bilayers are designated with large arrowheads; vesicles that contain intralumenal viral particles are denoted with small arrows. (B and C) Cells were transfected with a plasmid that expresses GFP-LC3 and subsequently infected with poliovirus for 5 h as in Figure 2. GFP-LC3 was visualized using a secondary antibody coupled to 10-nm gold particles; examples of such particles are denoted with arrows. An arrowhead identifies apparently extracellular packets of cytosol. (D) Cells transfected with an GFP-LC3-expressing plasmid and infected with poliovirus as in (C) were immunostained using an antibody directed against VP1, a viral capsid protein and visualized using a secondary antibody conjugated to 10-nm gold particles; examples of such particles are identified with arrows.

A second possibility for the greater reduction in extracellular than intracellular virus is that double-membraned structures with the cytoplasm provide a topologically reasonable mechanism for nonlytic release of cytoplasmic contents. Ultrastructural analysis studies of cells infected with poliovirus [1,2] and with rhinovirus 14 (Figure 8A) have revealed the presence of virions and other cytoplasmic material within the lumen of double-membraned vesicles. Presumably, these are double-membraned vesicles that formed relatively late in infection and were therefore able to trap viruses already present in the nearby cytoplasm. The known proximity of RNA replication, protein expression, and virion packaging to the membrane-associated replication complexes [59,60] should facilitate such events. Figure 8B displays an image consistent with the release of the packets of cytosol expected if the outer bilayer of a multilamellar membrane structure were to fuse with the plasma membrane. Figure 8C shows that, in poliovirus-infected cells, such blebs can contain LC3, and Figure 8D shows that they can also contain VP1, a viral capsid protein.

## Discussion

In this work, we have shown that several hallmarks of cellular autophagosomes, including the localization of GFP–LC3 into discrete punctate structures and staining with MDC followed by fixation, can be observed in human cells whether they are treated with tamoxifen or infected with poliovirus or rhinovirus. Furthermore, we have employed a new criterion for autophagy, the colocalization of LC3 and LAMP1, to demonstrate that this intracellular rearrangement was observed in cells treated with tamoxifen, infected with picornaviruses (poliovirus, rhinovirus 2, or rhinovirus 14) or transfected with plasmids that express poliovirus proteins 2BC and 3A (see Figures 2 and 8). Taken together with the previously observed double-membraned morphology, cytoplasmic contents, and complex origin of the membranous structures induced during poliovirus infection [1,2,35], we argue that the viruses utilize components of the autophagosome formation pathway to form the characteristic double-membraned vesicles seen during infection.

Do the autophagosome-like membranous structures induced by poliovirus act as scaffolds for RNA replication, or are they part of the host antiviral response? The numerous positive correlations between the functional presence of autophagosomal pathways and increased viral yield lead us to conclude that the autophagosome-like structures observed during poliovirus infection are not antiviral. Instead, we argue that the double-membraned vesicles induced during poliovirus infection facilitate poliovirus replication, and we hypothesize that poliovirus, rhinovirus 2, and rhinovirus 14 subvert the constituents of the cellular autophagy pathway to form membranous scaffolds on which RNA replication complexes can assemble.

Other positive-strand RNA viruses that have been shown to localize their RNA replication complexes to double-membraned vesicles in the cytoplasm of infected cells are equine artirivirus [61], murine hepatitis virus [62], and SARS virus [63]. The membranes associated with murine hepatitis virus infection have been shown recently to contain LC3 protein [64]. Strikingly, infection of murine ES cell lines deficient for APG5 with murine hepatitis virus was shown to result in a large decrease in the extracellular yield of this enveloped virus; the effect on intracellular viral particles or RNA was not reported. Nevertheless, the large reduction in yield of extracellular virus seen in the absence of Atg5p protein argues that this component of the cellular autophagy pathway is crucial for some step in the formation or egress of infectious virions of this murine coronavirus [64].

For both poliovirus and equine artirivirus, molecular inducers of double-membraned vesicle formation have been identified. Specifically, coexpression of poliovirus proteins 2BC and 3A is required to accumulate double-membraned vesicles [35] and to elicit the colocalization of GFP–LC3 and LAMP1 that correlates with the formation of autophagosomes (see Figure 3A). For equine artirivirus, the coexpression of viral proteins nsp2 and nsp3 is sufficient to induce the formation of double-membraned vesicles [65]. We anticipate that these viral proteins, likely to be capable of mimicking, intercepting, or corrupting the pathway of cellular autophagy, will prove to be useful tools to decipher its mechanism.

Recent work has highlighted an important role for autophagy in the innate immune response of vertebrates to intracellular pathogens. For example, induction of autophagy has been shown to promote clearance of Mycobacterium tuberculosis from infected macrophage [66]. Furthermore, during infection with *Shigella flexneri,* the wild-type function of the bacterial *icsB* gene was shown to be required to prevent autophagic degradation, a process that the authors argue is specifically induced by a bacterial protein, the product of the *virG* gene [67].

**Figure 9:**
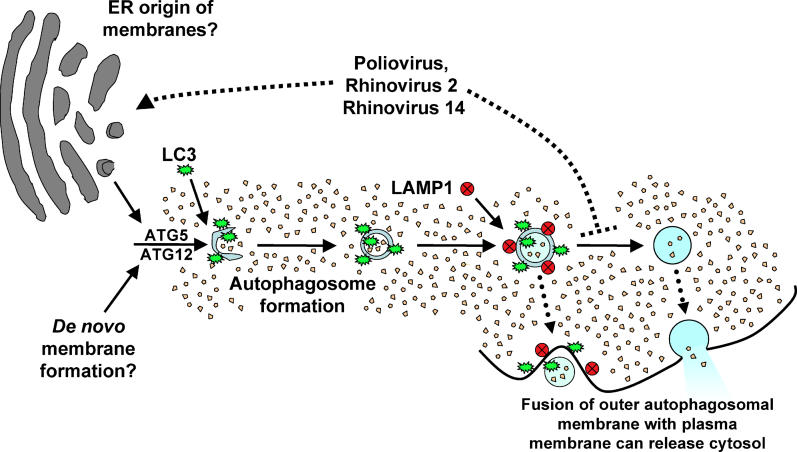
Pathway of Autophagosome Formation, Autophagic Degradation, and Proposed Steps of Pathway Subversion by Poliovirus and Related Viruses Double-membraned autophagosomes form either from ER membrane or de novo, encapsulating cytosol; the action of many gene products, including Atg5p and Atg12p, are required. LC3 protein (the Atg8p homolog) is associated with “sequestration crescents” as well as fully formed double-membraned autophagosomes. LAMP1 acquisition is a hallmark of the maturation of these structures, which eventually fuse with lysosomes to produce mature autophagosomes with single membranes and electron-dense contents. We hypothesize that infection by poliovirus or rhinovirus induces accumulation of autophagosomes to promote viral RNA replication by accelerating the formation of autophagosome-like structures from ER membranes, blocking the maturation of these structures into degradative organelles, or both (upper dotted line). The double-membraned topology makes the extracellular release of virions trapped in the cytosolic lumen topologically plausible, providing a mechanism for viral release in the absence of cell lysis. This could occur either from a double-membraned structure or from one in which only one of the membranes remained (dotted arrows).

Like *S. flexneri,* successful microorganisms often display strategies to evade potent host defenses. Furthermore, some microorganisms actively subvert otherwise effective host defense responses for their own benefit: for example, the growth of mink focus-forming virus requires apoptotic caspase activity for the maturation of a nonstructural protein [68], and murine cytomegalovirus encodes a chemokine homolog to attract cells of the immune system to the site of infection, which then promote viral dissemination (reviewed in [69]). Similarly, precedents are beginning to be established in which the autophagic pathway or its constituents may be subverted by intracellular pathogens to benefit their own replication (reviewed in [42,43]). As shown in Figure 9, the pathway of autophagosome formation ends with the acquisition of the lysosomal proteases and lipases that render the autophagosome a degradative organelle [27,28]. Mature autolysosomes are no longer bounded by double membranes because the inner membrane and luminal contents are degraded, becoming electron-dense and compact [24]. In *Legionella* infection, several genes, termed *Dot* or *Icm* genes, are required to retard the progression of autophagosome maturation, presumably to benefit bacterial growth within organelles that subvert components of the autophagosome [70,71,72]. We argue that poliovirus, rhinovirus 2, rhinovirus 14, equine encephalitis virus [65], and murine hepatitis virus [64] have similarly evolved a mechanism to accumulate autophagosome-like membranes in the cytoplasm for the duration of the infection. To maintain most of these structures in their double-membraned form, viral infection may both induce their formation and prevent their maturation into degradative organelles.

Why would a virus choose a double-membraned, autophagosome-like vesicle on which to replicate its RNA? Not all positive-strand RNA viruses utilize such structures. For example, Flock House virus RNA replication complexes assemble on outer mitochondrial membranes [73]. When the RNA replication complexes of a subgenomic Flock House virus RNA were experimentally redirected to the cytoplasmic surface of the ER of *S. cerevisiae,* the yield of replicated RNA actually increased [73]. Therefore, viral RNA replication displayed no mitochondrion-specific requirement for specific lipids, proteins, or processes. Presumably, however, there are reasons why particular positive-strand RNA viruses target particular intracellular membranes on which to assemble their replication complexes, some of which might not be assayed under single-cycle growth conditions in tissue culture.

A larger effect on extracellular than intracellular virus yield was observed when the abundance of autophagy proteins Atg12p and LC3 was reduced by RNAi (see Figure 7). Possible explanations for the observed preferential decrease in extracellular virions are that the reduction in autophagosome machinery decreased cell lysis early in infection, or that reduced abundance of autophagosomal machinery decreased nonlytic viral escape. Although our data do not yet distinguish between these hypotheses, we will discuss the latter possibility because nonlytic delivery of cytosol to the extracellular milieu could be a unique characteristic of multilamellar vesicles, and we were able to obtain ultrastructural images consistent with this interpretation (see Figure 8).

Nominally lytic viruses are often assumed to spread exclusively via cell lysis. However, the possibility of nonlytic viral release, even for nonenveloped viruses such as poliovirus, has been suggested from numerous reports of persistently infected cell lines that continuously secreted infectious particles [74,75,76,77]. Even more convincingly, when polarized Caco-2 cultures, growing as intact monolayers, as shown by resistance to the passage of dyes and electric current between the apical and basolateral surfaces, were infected with poliovirus, newly synthesized virus was shown to emerge from only the apical surface [78]. That the egress of this virus did not correspond to a detectable breach in the monolayer argued that a nonlytic, and polarized exit route of unknown origin had been utilized.

We suggest a potential mechanism for this nonlytic release of cytosolic viral particles via the formation of double-membraned vesicles throughout the course of infection. Early in infection, the double-membraned structures would entrap cytosol, but this cytosol would be free of virions. However, at later stages of infection, the cytosol trapped by newly generated double-membraned structures would often contain viral particles. Poliovirions and related enteroviruses are relatively resistant to the low pH and active proteolysis that would prevail within the lumen of these vesicles should they mature [79,80]. As depicted in Figure 9, the fusion of the outer membrane of an intact double-membraned structure would result in the release of membrane-bound packets of cytoplasm into the extracellular milieu, whereas fusion of the membrane of a mature autophagosome would result in the direct release of cytosolic contents, and incompletely resolved double-membrane structures would result in the formation of more complex topologies. The presence of both LC3 and poliovirus capsid protein VP1 in extracellular structures adjacent to poliovirus-infected cells (see Figure 8C and 8D) is consistent with the release of at least partially intact packets of cytoplasm during poliovirus infection. We speculate that the formerly intracellular membranes surrounding these packets of cytoplasm would be short-lived outside the cell, with free virus being the eventual result.

Recently, HIV has been shown to exit human macrophages via the fusion of multivesicular bodies with the plasma membrane, rather than by directly budding from the cell surface as in HIV-infected T cells [81,82,83]. HIV gag protein directs the targeting of HIV particles to multivesicular bodies via direct binding to cellular protein Tsg101 [84,85], a component of the ESCRT complex required for the sorting of proteins into endosomes [86,87,88]. Similarly, the intracellular formation of double-membraned vesicles in poliovirus-infected cells provides a topologically reasonable mechanism for the extracellular delivery of cytosolic contents in the absence of cell lysis. We speculate that a mechanism to spread virus within tissues of infected hosts without cell lysis could provide an advantage to those positive-strand viruses that subvert constituents of the cellular pathway of autophagosome formation.

## Materials and Methods

### 

#### Viruses, plasmids, and cells.

Poliovirus Mahoney type 1 was isolated following transfection with an infectious cDNA [89] and propagated as previously described [34]. “Tet-off” MCF7 cells were obtained from Clontech (Palo Alto, California, United States) and propagated in DMEM + 10% FBS; MCF7 cells are highly inducible for autophagosome formation in response to tamoxifen [9]. Poliovirus stocks were titered on both HeLa H1 and MCF7 cells. The multiplicities of infections indicated refer to titers on the appropriate cell lines. H1–HeLa cells were used due to their permissiveness for rhinovirus infection. Rhinovirus stocks were obtained from the American Type Culture Collection ( ATCC, Manassas, Virginia, United States). Virus stocks were prepared in H1–HeLa cells ( ATCC) grown in EMEM supplemented with 0.2 M HEPES and 0.1 M MgCl_2_, and viral titers were measured by TCID_50_ as described previously [90].

To construct the GFP–LC3 fusion protein-expressing plasmid, *LC3B* sequences were amplified by PCR from a Human Lung Library (ResGen, Carlsbad, California, United States) using targeted primers containing EcoRI sites ( ACTGAATTCCCATGCCGTCGGAGAAG and TTTGAATTCTTACACTGACAATTTCA). The *LC3A* coding region was then inserted into the EcoRI site of pEGFP-C3 (Clontech, Palo Alto, California, United States) to create an EGFP–LC3 fusion protein under the control of the CMV immediate-early promoter. Expression of poliovirus proteins 2BC and 3A was performed as described previously [35]. The 293T cells, which express SV40 T antigen, were used to facilitate expression from the SV40 promoter in these plasmids.

#### Immunofluorescence

Cells were fixed using freshly made 4% formaldehyde in PBS for 10 min at room temperature. Poliovirus protein 3A and cellular protein LAMP1 were visualized by indirect immunofluorescence. For visualization of poliovirus 3A protein in the presence of GFP–LC3, cells were washed twice in PBS and incubated in a PBS solution that also contained 0.5% saponin, 10 mM sodium azide, 0.125% BSA, 3A monoclonal tissue culture supernatant at a dilution of 1:30, and rhodamine-linked antimouse secondary antibody (SC-2084, Santa Cruz Biotechnologies, Santa Cruz, California, United States) at a dilution of 1:200. Cells were incubated at 4 °C for 45 min, washed twice with PBS, and placed under Vectashield mounting medium (Vector Laboratories, Burlingame, California, United States). Visualization of LAMP1 was performed using a monoclonal LAMP1 antibody (Transduction Laboratories, Lexington, Kentucky, United States) at a dilution of 1:200.

#### MDC staining and costaining.

MDC (Sigma, St. Louis, United States) was stored at −20 °C under desiccant. A fresh stock solution of 5 mM MDC was made in 1:1 DMSO/EtOH immediately prior to adding to cultures. At 1 h before fixation, fresh medium that contained either 10 μM MDC in DMSO/EtOH or an equivalent volume of DMSO/EtOH was added to the cells. The cells were then fixed using a freshly made 4% formaldehyde solution in PBS for 10 min at room temperature and imaging was performed immediately. To ensure that membrane vesiculation induced by methods other than the induction of autophagy did not show similar MDC staining patterns, Golgi vesiculation was induced with 5 μM ilimaquinone [91] or 5 μM nocodazole [92]; the disappearance of intact Golgi was confirmed using BODIPY-C_5_ ceramide (Molecular Probes, Eugene, Oregon, United States). Under these conditions, punctate MDC staining was not observed (data not shown).

#### Microscopy and deconvolution analysis.

Microscopic analysis was carried out on an Olympus IX70 at 100X magnification. Images were captured and deconvolved using SoftWorx 2.50 on an SGI Octane workstation. MDC staining was detected at 360 nm excitation/457 nm emission. GFP–LC3 expression was detected at 490 nm excitation/528nm emission. Rhodamine was detected at 555 nm excitation/617 nm emission. Individual images from each stack were saved as TIFF files and processed in Adobe Photoshop 7.0.

#### RNA interference to reduce intracellular concentrations of *LC3* and *ATG12*

siRNA SMARTpools, consisting of four RNA duplexes targeting the gene of interest, and a control siRNA targeting firefly luciferase, were purchased from Dharmacon (Lafayette, Colorado, United States). For *LC3,* both *LC3A* and *LC3B* RNAs [93] were targeted, bringing the total number of transfected duplexes to eight. Pools consisted of an equal amount of each duplex. The siRNA sequences are given as sense/antisense pairs. The siRNA sequences for *ATG12* were: GGGAAGGACUU ACGGAUGUUU/5′P-ACAUCCGUAAGUCCUUCCCUU; GAACACCAAGUUUCACUGUUU/5′P- ACAGU GAAACUUGGUGUUCUU; GCAGU AGAGCGAACACGAAUU/5′P-UUCGUGUUCGCUCUACUGCUU; and UGUU GCAGCUUCCUACUUCUU/5′P- GAAGU AGGAAGCU GCAACAUU. The siRNA sequences for *LC3A* were: GGACGGCUUCCUCUAUAUGUU/5′P-CAUAU AGAGGAAGCCGUCCUU; CGGUGAUCAU CGAGCGCUAUU/5′P-U AGCGCUCGAUGAU CACCGUU; ACAU GAGCGAGUUGGUCAAUU/5′P-UU GACCAACUCGCUCAUGUUU; and CGCCCAU CGCGGACAUCUAUU/5′P-UAGAUGU CCGCGAU GGGCGUU. The siRNA sequences for *LC3B* were: CAAAGUUCCUUGUACCUGAUU/5′P-U CAGGU ACAAGGAACUUUGUU; GAUAAU AGAACGAU ACAAGUU/5′P-CUUGUAUCGUUCUAUUAUCUU; GU AGAAGAUGU CCGACUUAUU/5′P-UAAGU CGGACAUCUUCUACUU; and AGGAGACGUU CGGGAUGAAUU/ 5′P-UUCAU CCCGAACGUCUCCUUU.

Cells were grown to densities of 1—5 × 10^5^ per 6-cm dish in 2.5 ml EMEM without antibiotics, and transfected using Lipofectamine 2000 (Invitrogen, Carlsbad, California, United States) according to manufacturer's instructions. For each 6-cm dish, 100 total pmol of pooled siRNA was diluted in 250 μl of serum-free OptiMEM medium (Invitrogen) and, separately, 5 μl of Lipofectamine 2000 was diluted in 250 μl of OptiMEM. After an incubation of 5 min at room temperature, the diluted RNA and Lipofectamine 2000 were combined and incubated for 20 min at room temperature. The 500-μl mixture was then added to each dish and gently rocked to spread the lipid–RNA complexes. Growth curves and immunoblots were performed 48 h after transfection. Total cell extracts were made using RSB-NP40 extraction buffer (10 mM Tris [pH 7.5], 10 mM NaCl, 1.5 mM MgCl_2_, 1% NP-40) supplemented with protease inhibitors (Roche, Mannheim, Germany). Extract was separated on a 15% Laemmli gel and transferred to a PVDF membrane for Western blotting. Anti-LC3 immunoblotting was performed using rabbit antibody raised commercially (AnaSpec, San Jose, California, United States) against a peptide comprising the first 16 amino acids of murine LC3 (MPSEKTFKQRRSFEQR). Anti-Atg12p and anti-GAPDH immunoblotting was performed using antibodies from Zymed (South San Francisco, California, United States) and Research Diagnostics (Flanders, New Jersey, United States), respectively. Antibodies were diluted 1:3,000 in a PBS solution that also contained 0.1% Tween-20 and 2% BSA, and detected with alkaline-phosphatase conjugated goat antirabbit antibody, at a dilution of 1:10,000 using the ECF reagent from Amersham Biosciences (Piscataway, New Jersey, United States).

#### High-pressure freezing, freeze-substitution, and electron microscopy (EM).

For cryofixation and EM analysis, H1–HeLa cells were grown in EMEM supplemented with 0.2 M HEPES and 0.1 M MgCl_2_ in flasks. Cells were infected with virus at an MOI of 50 PFU/cell for poliovirus or 50 TCID_50_/cell for rhinovirus, then washed three times with PBS, trypsinized, and collected by centrifugation. The cell pellet was resuspended in 0.15 M mannitol in PBS and collected by centrifugation. Aliquots of the resulting pellet were frozen in a Balzers HPM 10 high-pressure freezing apparatus as described previously [2] and stored in liquid nitrogen. To observe cellular ultrastructure, samples were freeze-substituted in 0.1% tannic acid in acetone at −80 °C, rinsed in acetone, then warmed at −20 °C in the presence of 2% osmium tetroxide in acetone for 16 h, followed by incubation at 4 °C for 4 h. After rinsing in acetone at 4 °C, samples were embedded in Epon-Araldite resin. Thin sections were stained with 2% uranyl acetate and lead citrate and imaged at 80 kV in a JEOL 100C or Philips CM10 EM. For immunostaining, high-pressure frozen samples were freeze-substituted in 0.1% glutaraldehyde-0.05% uranyl acetate in acetone, embedded, stained, and visualized as described previously [35].

#### Accession Numbers

The SwissProt (http://us.expasy.org/sprot/) accession numbers for the gene products discussed in this article are 2BC and 3A (P03299), Atg12p (O94817), Atg8p (P38182), LAMP1 (P11279), LC3 (Q9GZQ8), mTor (P42345), Sec12p (P11615), Sec13p (P5573), Sec16p (P48415), Sec23p (P15303), Sec24p (P40482), and Sec31p (O94979).

## Abbreviations

ER: endoplasmic reticulum
GFP: green fluorescent protein
MDC: monodansylcadaverine
siRNA: small interfering RNA
